# *Spiraea prunifolia* var. *simpliciflora* leaves ameliorate inflammatory responses and oxidative stress in PPE/LPS-induced chronic obstructive pulmonary disease mouse model

**DOI:** 10.3389/fphar.2025.1501731

**Published:** 2025-04-03

**Authors:** Ba-Wool Lee, Ji-Hye Ha, Da-Hye Yi, Ju-Hong Kim, Seong-Hun Jeong, Hyeon Jin Lee, Yun-Hye Kim, Hyung-Jun Kwon, Ji-Young Park, Woo Sik Kim, Young-Bae Ryu, In-Chul Lee

**Affiliations:** ^1^ Functional Biomaterial Research Center, Korea Research Institute of Bioscience and Biotechnology, Jeongeup-si, Jeollabuk-do, Republic of Korea; ^2^ College of Veterinary Medicine and BK21 FOUR Program, Chonnam National University, Gwangju, Republic of Korea; ^3^ College of Veterinary Medicine and BK21 FOUR Program, Chungnam National University, Daejeon, Republic of Korea; ^4^ Center for Companion Animal New Drug Development, Korea Institute of Toxicology (KIT), Jeongeup-si, Jeollabuk-do, Republic of Korea

**Keywords:** *Spiraea prunifolia* var. *simpliciflora*, airway inflammation, oxidative stress, NOD-like receptor pyrin domain-containing 3 inflammasome, thioredoxin-interacting protein

## Abstract

*Spiraea prunifolia*: var. *simpliciflora* (SP) is a known medical food that is used to treat emesis, malaria, and fever. This study investigated the therapeutic potential of SP leaf extract on oxidative stress and airway inflammation using a chronic obstructive pulmonary disease (COPD) mouse model induced by porcine pancreatic elastase (PPE) and lipopolysaccharide (LPS). Male C57BL/6N mice were treated intratracheal instillation of PPE (0.05 units/50 μL) and LPS (5 μg/50 μL), and administered positive control (dexamethasone; 3 mg/kg) and SP (50 and 100 mg/kg). SP treatment decreased T helper type 1 (Th-1) cytokines as well as counts of macrophage and neutrophil in bronchoalveolar lavage fluid of PPE/LPS-induced COPD. SP treatment reduced alveolar destruction, inflammatory cell infiltration, and collagen fiber with improvement of forced expiratory volume to forced vital capacity ratio and lung elastance in lung tissue. SP downregulated thioredoxin-interacting protein and NOD-like receptor pyrin domain-containing 3 inflammasome which inhibited caspase-1 and IL-1β expression. SP attenuated production of reactive oxygen and nitric oxide through enhancement of nuclear factor-erythroid 2-related factor translocation with elevation of heme oxygenase-1 and NAD(P)H quinone oxidoreductase 1 expression. Furthermore, SP attenuated the levels of reactive oxygen species and nitric oxide in mice with PPE/LPS-induced COPD. Thus, SP has the therapeutic potential for COPD treatment.

## 1 Introduction

Chronic obstructive pulmonary disease (COPD) is a persistent inflammatory lung condition that is currently listed as the third leading cause of mortality and morbidity worldwide (World Health Organization, 2018). The prominent pathological feature of COPD is airflow limitation and airway inflammation with emphysema which is induced by exposure to air pollutants, including industrial particles, gases, and cigarette smoking (Boskabady and Mhtaj, 2014; Vogelmeier et al., 2017). Air pollutants and cigarette smoking can generate reactive oxygen species (ROS), mucus production, and pro-inflammatory cytokines contributed to the activation of inflammatory cells. Activated inflammatory cells, particularly macrophages and neutrophils, can produce T helper type 1 (Th-1) cytokines, including interleukin (IL)-1β, IL-6, and tumor necrosis factor (TNF)-α. Th-1 cytokines are associated with various pathophysiological alterations, such as mucus hypersecretion, airway inflammation, and fibrosis, leading to bronchiolar obstruction (Ko et al., 2019; Tang et al., 2023).

Oxidative stress is related to an imbalance in oxidant/antioxidant molecules and is the outcome of excessive ROS production. ROS-mediated oxidative stress causes extracellular matrix remodeling, alveolar cell injury, and bronchiolar obstruction, all of which contribute to the development of COPD (Wang et al., 2020; Tian et al., 2021). Nuclear factor-erythroid 2-related factor 2 (Nrf-2) defends damages caused oxidative stress in cells and tissues (Wang et al., 2019). Upon cellular oxidative conditions, Nrf-2 separates from Kelch-like ECH-associated protein 1 (Keap-1) and attaches to antioxidant response elements (AREs) in the nucleus, resulting in the expression of diverse antioxidant enzymes, including NAD(P)H quinone dehydrogenase 1 (NQO1) and heme oxygenase 1 (HO-1). HO-1 degrades heme into biliverdin and bilirubin, which possesses antioxidant properties, whereas NQO1 eliminate ROS and NO production by reducing of quinones (Huang et al., 2020; Tonolo et al., 2022). Therefore, upregulation of the Nrf-2/HO-1/NQO1 pathway is considered an important therapeutic target for COPD progression.

Thioredoxin (TXN) and thioredoxin-interacting protein (TXNIP) are reduction-oxidation signaling complex that play crucial roles in oxidative stress- and inflammation-mediated diseases, such as diabetes, autoimmune disease, cancer, and apoptosis (Kansal et al., 2023; Qayyum et al., 2021). TXN, a redox state-controlled regulator, can decrease oxidative stress in the intracellular environment via activating disulfide reductase. TXNIP interacts with TXN directly and inhibits its reducing activity and expression (Qayyum et al., 2021). ROS facilitates TXN-TXNIP dissociation and the dissociated TXNIP interacts with NOD-like receptor pyrin domain-containing 3 (NLRP3), resulting in inflammasome activation (Wang et al., 2020; Zhang Q. et al., 2021). NLRP3 inflammasome consists of NLRP3 oligomers, effector pro-caspase-1, and apoptosis-associated speck-like protein (ASC) (Mahalanobish et al., 2020). NLRP3 inflammasome triggers the proteolytic cleavage of caspase-1, which activates pro-IL-18 and pro-IL-1β via transformation to their active forms, promoting inflammation. (Tian et al., 2021). ROS-mediated activation of the TXNIP/NLRP3 inflammasome is related to lung inflammatory responses elicited during COPD progression (Colarusso et al., 2017; Qayyum et al., 2021). Therefore, suppression of the TXNIP/NLRP3 inflammasome may contribute to anti-oxidant and anti-inflammatory responses necessary in attenuating of COPD.

*Spiraea prunifolia* var. *simpliciflora* (SP) is found throughout the Republic of Korea and is traditionally used in medicine to treat emetic conditions, fever, and malaria (Oh et al., 2001; So et al., 1999). In previous studies, SP exerted anti-inflammatory and antioxidant activities via inhibiting Th-1 cytokine production and downregulating nitric oxide and superoxide levels in lipopolysaccharide (LPS)- or polymyristic acetate-stimulated RAW264.7 cells, respectively (Kim et al., 2017a; So et al., 1999). The ethanol fraction of SP inhibited nitric oxide synthase (iNOS), Th-1 cytokines, and cyclooxygenase (COX)-2 via inhibition of NF-κB phosphorylation in LPS-stimulated RAW264.7 cells (Suhr et al., 2022). However, the activities of the SP methanol extract on oxidative stress and inflammatory responses in COPD have not been evaluated. Thus, we aimed to investigate the protective effects of SP against porcine pancreatic elastase (PPE)/LPS-induced COPD in a murine model.

## 2 Materials and methods

### 2.1 Plant material and UPLC-QTOF-MS analysis

The SP extract (KPM040-068) were obtained from the Natural Product Central Bank at the Korea Research Institute of Bioscience and Biotechnology (KRIBB, Republic of Korea). The SP leaves dried under shade for 2 weeks and were extracted in enclosed ultrasonic extractor (SDN-900H, SD-ULTRASONIC CO., LTD.) using methyl alcohol 99.9% (HPLC grade) as the extract solvent. The ultrasonic extractor conditions were set up as follow: 1,500 W, 40 KHz, ultrasonication 2 h, 30 cycles. After the extraction process, mixture was filtered using a qualitative filter (HYUNDAI MICRO CO., LTD.) at room temperature and drying under reduced pressure. SP analysis was performed on an ACQUITY UPLC system coupled with Vion IMS QToF mass spectrometer (UPLC-QToF-MS; Waters, MA, United States), equipped with a BEH C18 column (1.7 μm, 2.1 × 100 mm) at 35°C and 10°C, respectively. The mobile phases consisted of 0.1% formic acid in water and acetonitrile (a), using a gradient from as follows: 0–2 min, 5% a; 2–12 min, 5%–30% a; 12–15 min, 30%–100% a. The flow rate was 0.4 mL/min and the injection volume was 2 μL. The mass spectrometer conditions were as follow: desolvation temperature, 350°C; source temperature, 110°C; desolvation gas (N_2_) flow rate, 800 L/h; capillary voltage, 2,000 V; cone voltage, 50 V. The mass analyzer operated in positive mode, scanning over a mass range of 100–1,500 Da a scan time of 0.2 s. Data acquisition was carried out by external reference (Lock-Spray™).

### 2.2 Chemicals and materials

RAW264.7 cell were purchased from Korean Collection for Type Culture (Seoul, Republic of Korea). LPS, PPE, tribromoethanol (Avertin), and dexamethasone (DEX) were obtained from Sigma-Aldrich (MO, United States). The ROS detection assay kit (DCF-DA; CellRox^®^ green reagent; Thermo Scientific, MA, United States), reduced glutathione (GSH) activity assay kit, thiobarbituric acid-reactive substances (TBARS; DoGen, Seoul, Republic of Korea), nitric oxide plus kit (iNtRON Biotechnology, Republic of Korea), ELISA kits for TNF-α, IL-6, and IL-1β (R&D system, MN, United States), and Diff-Quik^®^ Stain kit (IMEB, CA, United States) were used according to the manufacturer’s instructions.

### 2.3 Cell culture and cytotoxicity

The RAW264.7 cells were cultured in Dulbecco modified Eagle medium supplemented with 10% heat-inactivated fetal bovine serum and 1% antibiotics in a 5% CO_2_ incubator (37°C). The RAW264.7 cells (5 × 10^4^ cells/well) were grown in 96-well plates for 24 h. After 24 h, cells were incubated with different concentrations of SP (0, 100, 50, and 25 μg/mL) for 24 h. The plate incubated with EZ-CyTox (DoGen) for 1 h and the absorbance was conducted at 450 nm.

### 2.4 Measurement of Th-1 cytokine levels and oxidative stress marker in LPS-stimulated RAW264.7 cells

The RAW264.7 cells (5 × 10^5^ cells/well) were seeded on 6-well plate and treated with various concentration of SP for 1 h before LPS (0.5 μg/mL). After 24 h, culture supernatants were collected to conduct the levels of Th-1 cytokine using a competitive ELISA kit (R&D system). NO and ROS production were evaluated using a nitric oxide plus kit (iNtRON Biotechnology) and CellRox^®^ green reagent (Thermo Scientific), respectively, according to the manufacturer’s instructions.

### 2.5 Immunoblotting and immunofluorescence in LPS-stimulated RAW264.7 cells

The RAW264.7 cells were cultured on 6-well plates and treated with various concentration of SP for 1 h before 0.5 μg/mL LPS. After incubation for 2 h, plates were washed with PBS and were lysed in M-PER Mammalian Protein Extraction Reagent containing protease and phosphatase inhibitor cocktail (Thermo Scientific). The protein (30 μg) was separated on a 4%–12% SDS-polyacrylamide gel and then transferred to a polyvinylidene fluoride (PVDF) membrane. The membranes were blocked by blocking solution (Thermo Scientific) and incubated with primary antibodies: caspase-1, IL-1β, NQO1, HO-1, and Nrf-2 (Abcam, Cambridge, United Kingdom), TXNIP (Novus Bio, Centennial, CO, United States), NLRP3 and β-actin (Cell Signaling Technology), α-tubulin and Lamin B (Thermo Scientific). After incubation for 24 h, membranes were washed with TBST washing buffer followed by incubation with 0.1 μg/mL secondary antibody (Cell Signaling Technology) for 1 h. Next three washes with TBST, bands were visualized with an enhanced chemiluminescence kit (Thermo Scientific) and were evaluated using chemiluminescent scanner (LI-COR, Biosciences, Lincoln, NE, United States).

For immunofluorescence analysis of Nrf-2, RAW264.7 cells were seeded on 8-well chamber slides (NUNC, Roskilde, Denmark) and treated with SP for 1 h prior to exposure to 0.5 μg/mL LPS. The slides were fixed with acetone solution (80%) for 15 min, washed with PBS, and incubated at 37°C for 1 h with Nrf-2 antibody (1:100 dilution, Abcam). After washing with PBS, the slides were treated with FITC-conjugated goat anti-mouse IgG antibody (1:100 dilution, Santa Cruz Biotechnology). Subsequently, the slides were rinsed with PBS, mounted using SlowFade™ Gold Antifade Mountant containing 4,6-diamidino-2-phenylindole (DAPI; Thermo Scientific), and visualized under a Leica TCS SP5 AOBS laser scanning confocal microscope (Leica Microsystems).

### 2.6 Experimental procedure

C57BL/6N male mice were purchased from Orient Bio (Republic of Korea) and handled as per the Institutional Animal Care and Use Committee of the KRIBB (Approval number: KRIBB-AEC-20113). The mice were split into five groups (*n* = 7). The animals were treated on PPE (days 0 and 7; intratracheally instillation of 0.05 units) and LPS (days 4 and 11; intratracheally instillation of 5 μg) in 50 μL of phosphate-buffered saline (PBS; Lonza, Switzerland) while the mice were under anesthesia, using an automatic video instillator (Kim et al., 2018). The PPE/LPS-induced COPD model was developed using the method by Kim et al. (2022). Mice were administered with SP (50 and 100 mg/kg) and positive control (DEX; 3 mg/kg) from days 4 to day 11 (Kim et al., 2017b).

### 2.7 Pulmonary function analysis

To analyze pulmonary function, mice were anesthetized by 2.5% avertin (Sigma-Aldrich, intraperitoneal injection; 0.02 mL/g weight) and a tracheostomy was performed. Pulmonary function was established using a flexivent (SCIREQ Scientific Respiratory Equipment Inc., Canada). The lung volume was evaluated with deep inflations (6 s) and total elastance and tissue elastance were conducted by snapshot and Quick Prime-3, respectively (Li et al., 2020). The forced expiratory volume (0.1 s; FEV_0.1_) and forced vital capacity (FVC) were evaluated by Negative pressure-driven forced expiration (NPFE) maneuver and the FEV_0.1_/FVC ratio was calculated using flexivent software (Shalaby et al., 2010).

### 2.8 Bronchoalveolar lavage fluid (BALF) analysis

BALF was collected as per the method by Shin et al. (2015). To determine various cell counts, BALF was centrifuged at 800 rpm, 10 min and the supernatants were stored at −70°C for analysis of cytokines. The pellets were re-suspended with PBS (1.4 mL) and attached to the slide using cytospin (Cytospin 4 centrifuge, Thermo Scientific) (800 rpm, 5 min, 20°C). Total cell numbers and types of inflammatory cells were assessed on a Diff-Quik^®^ staining reagent. Thereafter, macrophages, lymphocytes, neutrophils, and total cells were counted. In the BALF, Th-1 cytokine levels were determined by ELISA (R&D system) and evaluated using a plate reader (450 nm; Bio-Rad Laboratories, United States).

### 2.9 Histopathological analysis

The lung tissue samples were fixed in 10% phosphate-buffered formalin, embedded in paraffin wax, and sectioned into 4 μm slice. These slices were stained with H&E (BBC Biochemical, United States). The stained samples were then examined under a light microscope (Leica Microsystems) to assess airway inflammation, mean alveolar number (MAN), and mean linear intercept (MLI). The degree inflammation each slide was graded (0, no lesions; 1, minimal; 2, mild; 3, moderate; and, 4, severe). MLI was used to conduct the average distance between alveolar wall and MAN provided a quantitative estimation of alveolar density (Wang et al., 2024). To evaluate MAN and MLI, the number of intercepts (NS) and total alveolar count (Na) were recorded to determine the image area (S) and total length of lines (L) (Geng et al., 2016; Wang et al., 2024). The calculations were performed using the formulas MLI = L/NS and MAN = Na/S. The average value for each indicator was determined from seven randomly chosen fields and used for statistical analysis. Masson’s trichrome stain (Abcam) was performed via kits and used to quantify collagen fibers. Masson’s trichrome stain was measured as described in a previous study (Cheng et al., 2024).

### 2.10 Immunoblotting in lung tissues

Lung tissue samples were separated to nuclear and cytoplasmic proteins using NE-PER Nuclear and Cytoplasmic Extraction Reagents (Thermo Scientific) following the manufacturer’s instruction. The levels of Nrf-2/HO-1/NQO1 and β-actin expression were conducted by immunoblotting as described above.

### 2.11 Measurement of oxidative stress markers

The lung tissues were homogenized in a PBS and centrifuged at 14,000 rpm for 15 min at 4°C to collect supernatants. The lung tissues were collected and analyzed ROS and NO production according to the manufacturer’s instructions as described above. The contents of glutathione (GSH) and thiobarbituric acid-reactive substances (TBARS), a marker of lipid peroxidation, were determined by a commercial assay kit (DoGen) in obtained supernatants according to the manufacturer’s instructions.

### 2.12 Statistical analyses

All values are presented as the means ± standard deviation (SD). Statistical evaluation was conducted using analysis of variance, followed by a Tukey’s multiple comparison test. P values ≤0.05 and ≤0.01 were considered to be statically significant. All analyses were performed using the GraphPad Software (CA, United States).

## 3 Results

### 3.1 Bioactive components in SP extract

**FIGURE 1:**
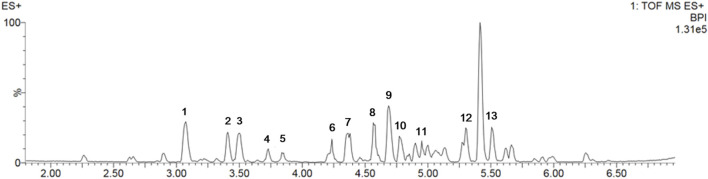
The metabolites contained in SP were analyzed in positive mode using ultra-performance liquid chromatography-quadrupole-time of flight mass spectrometry (UPLC-QToF-MS).

The components of *S. prunifolia* var. *simpliciflora* were evaluated using UPLC-Q-TOF MS. The linear relationships and detection limit of each component are shown in Figure 1, and results displayed that SP contained 1-O-caffeoyl-β-D-glucopyranoside, 1-O-caffeoylquinic acid, 4-O-β-D-glucopyranosyl-cis-cinnamic acid, meliadanoside B, apocynoside I, quercetin-3-O-β-D-xylo-pyranosyl-(1→2)-β-D-glucopyranoside, (−)-chebulic acid triethyl ester, robinetinm, neocomplanoside, ginkgolide B, 5,7,2',5'-tetrahydroxy-flavone, skimmin, and smyrindioloside, which mainly flavonoids and polyphenols as presented in Table 1.

**TABLE 1:** Tentatively identification of major peaks detected in *Spiraea prunifolia* var. *simpliciflora*.

	Peak No.	RT (min)	Identification	Adducts	Exact mass (m/z)	Mass error (ppm)	Fragment ions (m/z)
ES+	1	3.07	1-O-Caffeoyl-β-D-glucopyranoside	[M + Na]^+^	365.0838	−1.4	181, 163, 145
	2	3.41	1-O-Caffeoylquinic acid	[M + Na]^+^	377.0836	−2.0	163, 145
	3	3.50	4-O-β-D-Glucopyranosyl-cis-cinnamic acid	[M + Na]^+^	349.0879	−4.2	165, 147
	4	3.73	Meliadanoside B	[M + Na]^+^	351.1041	−2.5	163, 145
	5	3.84	Apocynoside I	[M + Na]^+^	409.1822	−2.7	163
	6	4.24	Quercetin-3-O-β-D-xylopyranosyl-(1→2)-β-D-glucopyranoside	[M + H]^+^	597.1442	−1.4	495, 303, 145
	7	4.36	(−)-Chebulic acid triethyl ester	[M + Na]^+^	463.1204	−1.5	261
	8	4.56	Robinetin	[M + H]^+^	303.0497	−0.7	229, 153
	9	4.69	Neocomplanoside	[M + Na]^+^	527.1152	−1.4	303, 295, 163, 145
	10	4.77	Ginkgolide B	[M + Na]^+^	447.1253	−1.8	283
	11	4.95	5,7,2',5'-Tetrahydroxy-flavone	[M + H]^+^	287.0547	−1.2	147
	12	5.30	Skimmin	[M + H]^+^	325.0916	−0.5	307, 163, 145
	13	5.51	Smyrindioloside	[M + H]^+^	423.1282	−0.8	287, 163

### 3.2 Effects of SP on Th-1 cytokine in LPS-stimulated RAW264.7 cells

**FIGURE 2:**
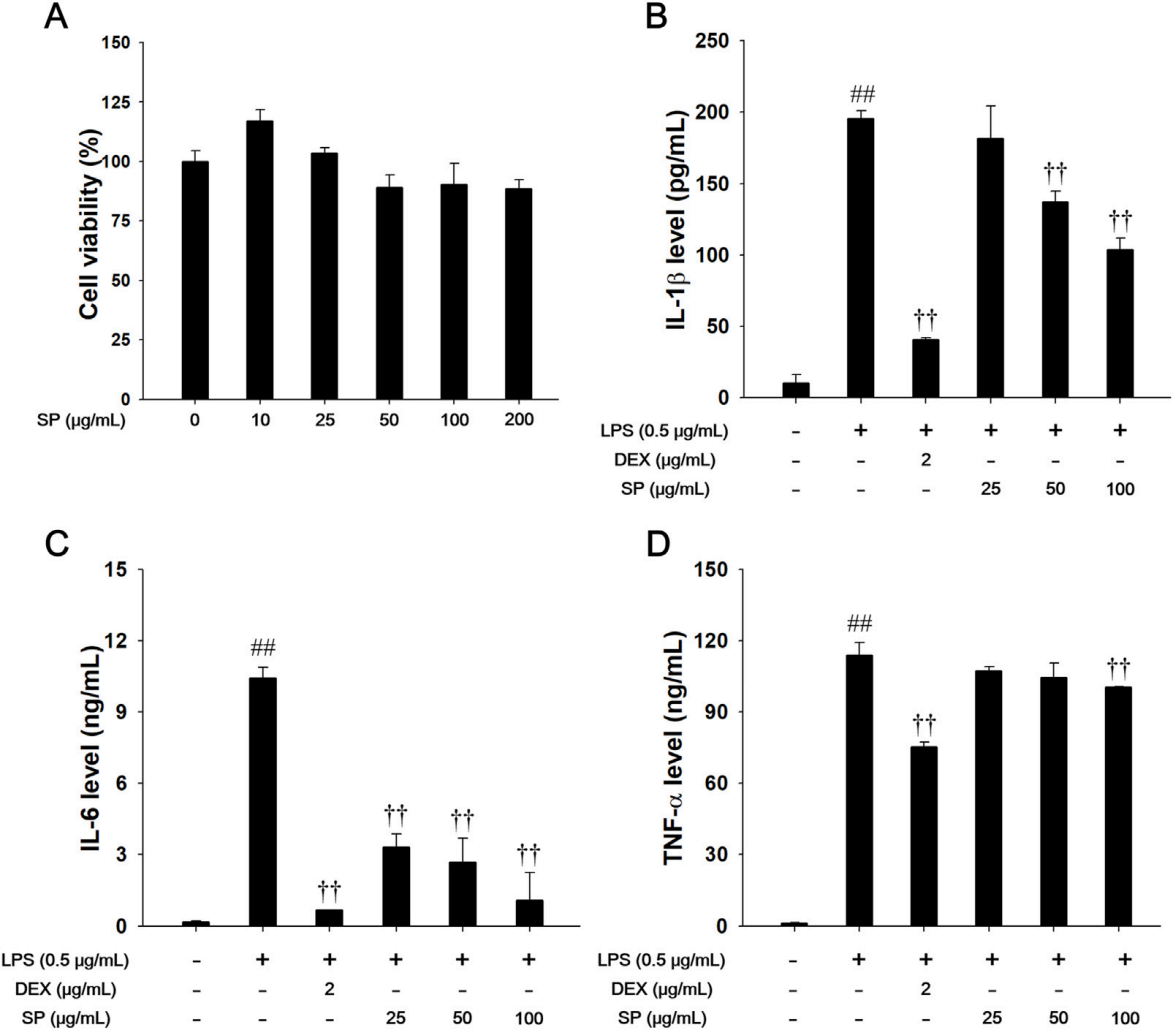
Effects of SP on Th-1 cytokines in LPS-stimulated RAW264.7 cells. **(A)** The cell viability was measured using a WST-1 reagent, and SP was treated with 10, 25, 50, 100 and 200 μg/mL for 24 h. The production of **(B)** IL-1β, **(C)** IL-6, and **(D)** TNF-α was determined by ELISA. The culture medium was changed DMEM (0.1% FBS) and treated with SP (25, 50 and 100 μg/mL) for 1 h and incubated with LPS (0.5 μg/mL) for 24 h (IL-1β, IL-6, and TNF-α). NC: Non-stimulated cells; LPS: LPS-stimulated RAW264.7 cells; DEX: dexamethasone (2 μg/mL) + LPS-stimulated RAW264.7 cells; SP: SP (25, 50 and 100 μg/mL) + LPS-stimulated RAW264.7 cells. The values are expressed as the means ± SD (*n* = 3). ^##^ *P* < 0.01, significantly different from control; ^††^ *P* < 0.01, significantly different from LPS-stimulated RAW264.7 cells.

Different SP concentrations were used in this experiment (Figure 2A). LPS (0.5 μg/mL) treatment markedly increased the levels of Th-1 cytokines (Figures 2B–D). In contrast, SP-treated cells showed lower level of Th-1 cytokines than LPS-stimulated cells, in a concentration-dependent manner.

### 3.3 Effects of SP on Nrf-2 pathways, ROS and NO production in LPS-stimulated RAW264.7 cells

**FIGURE 3:**
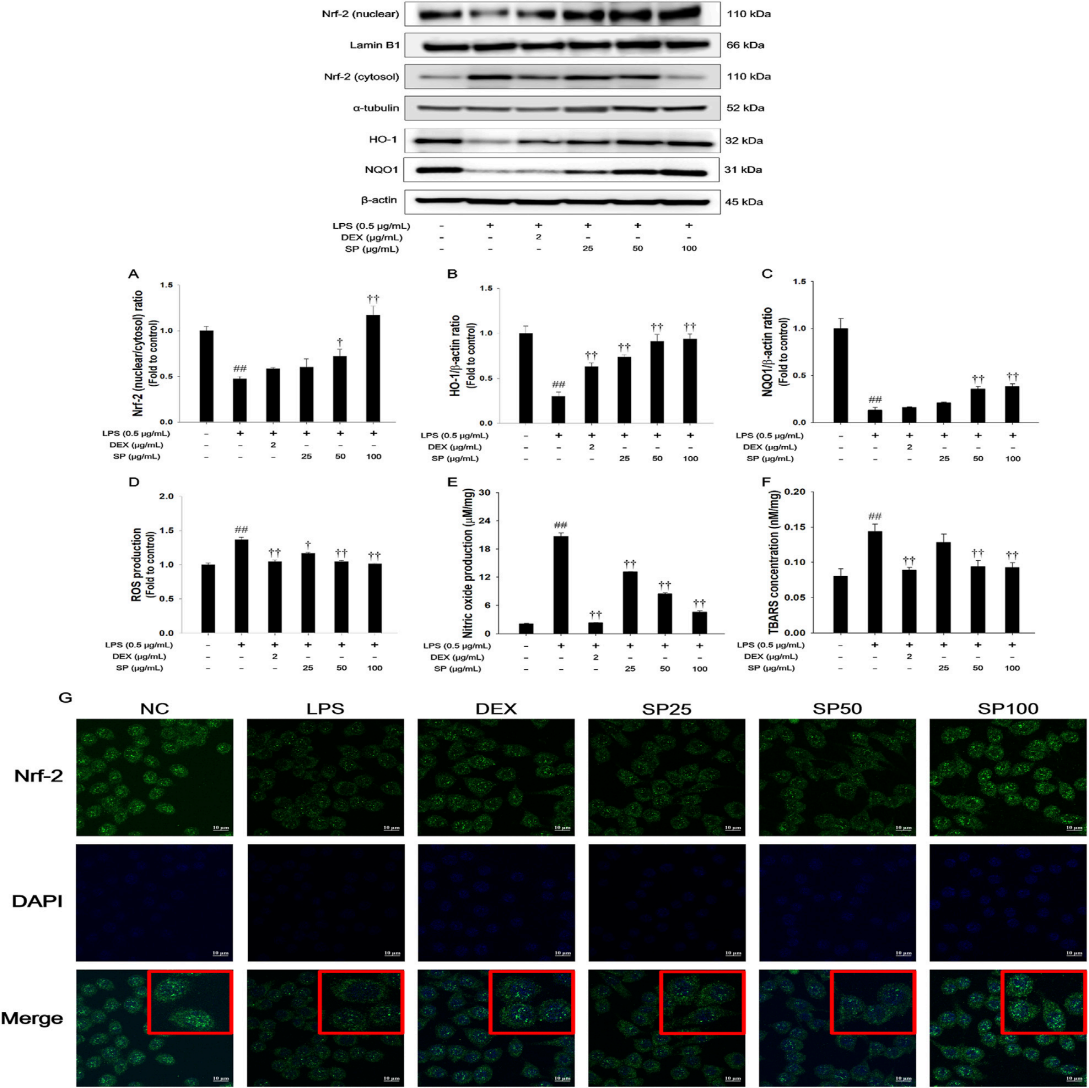
Effects of SP on Nrf-2/HO-1/NQO1 pathway and oxidative marker in LPS-stimulated RAW264.7 cells. The **(A)** Nrf-2, **(B)** HO-1, and **(C)** NQO1 were analyzed by Western blot (Lamin B1, α-tubulin, and β-actin: loading control). The **(D)** ROS, **(E)** NO, and **(F)** TBARS contents were measured in LPS-stimulated RAW264.7 cells. **(G)** Nrf-2 in RAW264.7 cells were determined by immunofluorescence analysis. The culture medium was changed DMEM (0.1% FBS) and treated with SP (25, 50 and 100 μg/mL) for 1 h and incubated with LPS (0.5 μg/mL) for 2 h (Nrf-2, HO-1, NQO1, and TBARS) and 24 h (ROS and NO production). NC: Non-stimulated cells; LPS: LPS-stimulated RAW264.7 cells; DEX: dexamethasone (2 μg/mL) + LPS-stimulated RAW264.7 cells; SP: SP (25, 50 and 100 μg/mL) + LPS-stimulated RAW264.7 cells. The values are expressed as the means ± SD (*n* = 3). ^##^ *P* < 0.01, significantly different from control; ^†,††^ *P* < 0.05, *P* < 0.01, significantly different from LPS-stimulated RAW264.7 cells.

As shown in Figure 3, LPS treatment resulted in decreased nuclear translocation of Nrf-2 and inhibited the expression of HO-1/NQO1 in LPS-stimulated RAW264.7 cells (Figures 3A–C). In contrast, SP treatment significantly enhanced nuclear translocation of Nrf-2 with increased HO-1/NQO1 expression. Moreover, SP treatment notably suppressed levels of ROS, NO, and TBARS (Figures 3D–F). Based on the immunofluorescence analysis of Nrf-2 (Figure 3G), LPS treatment decreased Nrf-2 while SP markedly increased nuclear translocation of Nrf-2 in the RAW264.7 cells in a concentration-dependent manner.

### 3.4 Effects of SP on TXNIP/NLRP3 inflammasome in LPS-stimulated RAW264.7 cells

**FIGURE 4:**
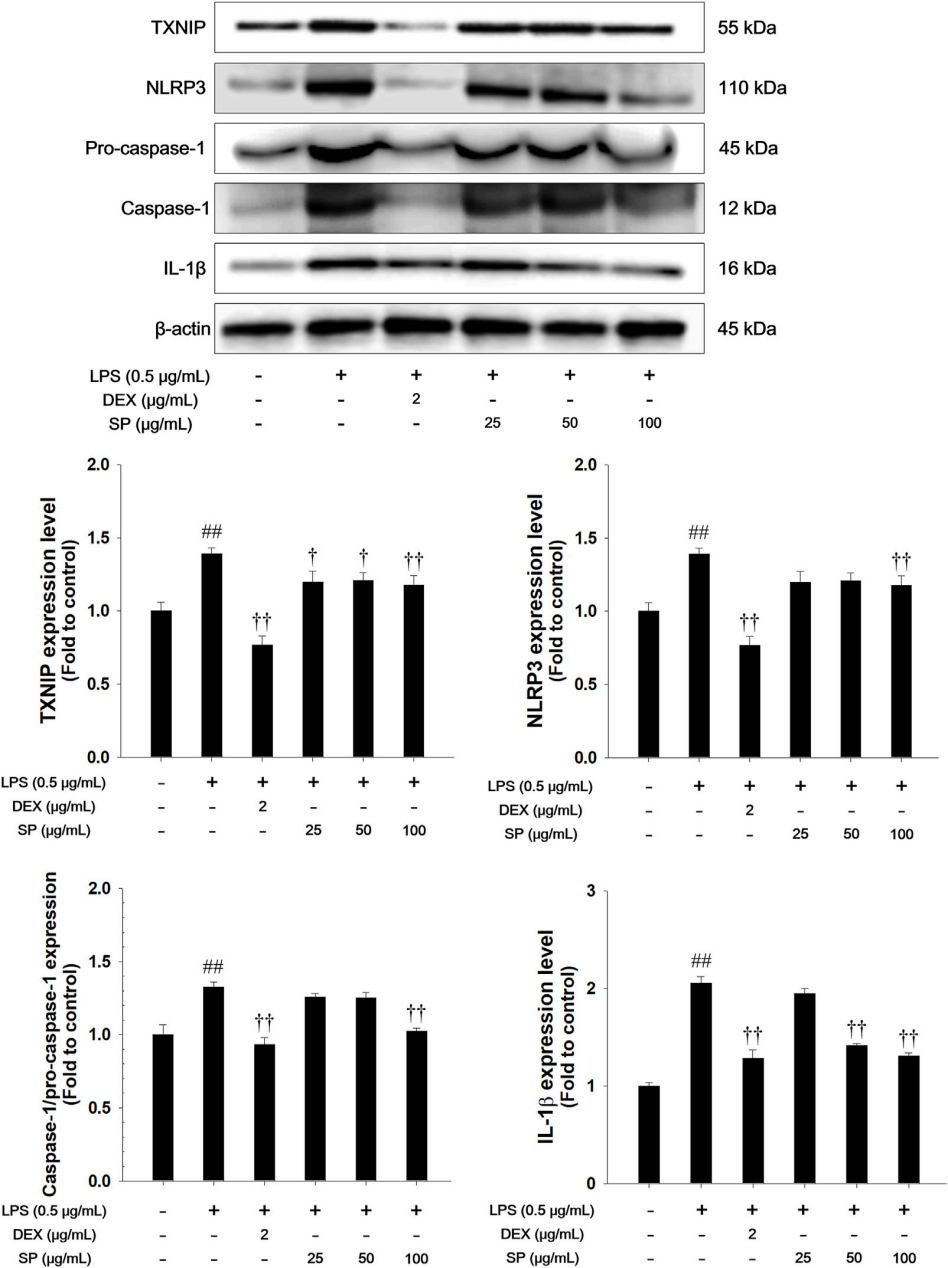
Effects of SP on TXNIP/NLRP3 inflammasome in LPS-stimulated RAW264.7 cells. The expression of TXNIP, NLRP3, pro-caspase-1, caspase-1, and IL-1β were analyzed by Western blot (β-actin: loading control). The culture medium was changed DMEM (0.1% FBS) and treated with SP (25, 50 and 100 μg/mL) for 1 h and incubated with LPS (0.5 μg/mL) for 30 min (NLRP3), 6 h (TXNIP, pro-caspase-1, and caspase-1), and 24 h (IL-1β), respectively. NC: Non-stimulated cells; LPS: LPS-stimulated RAW264.7 cells; DEX: dexamethasone (2 μg/mL) + LPS-stimulated RAW264.7 cells; SP: SP (25, 50 and 100 μg/mL) + LPS-stimulated RAW264.7 cells. The values are expressed as the means ± SD (*n* = 3). ^##^ *P* < 0.01, significantly different from control; ^†,††^ *P* < 0.05, *P* < 0.01, significantly different from LPS-stimulated RAW264.7 cells.

The LPS-stimulated RAW264.7 cells showed a remarkable increase in the expression of TXNIP and NLRP3 concurrent with elevated active forms of caspase-1 and IL-1β compared with non-stimulated cells (Figure 4). In contrast, treatment with SP significantly downregulated the levels of TXNIP and NLRP3 and inhibited the expression of caspase-1 and IL-1β in the LPS-stimulated RAW264.7 cells.

### 3.5 Effects of SP on inflammatory cell counts and Th-1 cytokine levels in BALF of the PPE/LPS-induced COPD model

**FIGURE 5:**
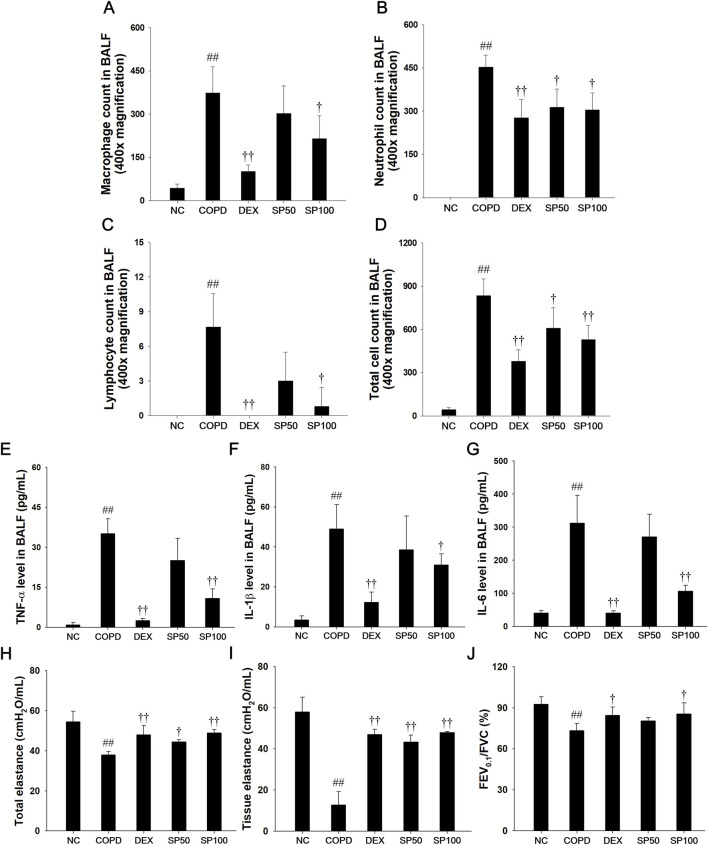
Effects of SP on inflammatory cell counts, Th-1 cytokines, and pulmonary function in PPE/LPS-induced COPD model. **(A–D)** The inflammatory cells were stained with Diff-Quik stain reagent. The levels of **(E)** TNF-α, **(F)** IL-1β and **(G)** IL-6 in BALF were determined by ELISA. **(H)** Snapshot perturbation maneuver measured the value of total elastance in the respiratory system **(I)** The tissue elastance were measured by Quick prime-3 maneuver. **(J)** Forced expiratory volume at 0.1 s (FEV_0.1_), forced vital capacity (FVC), and FEV_0.1_/FVC ratio were measured in mice by negative pressure-driven forced expiration maneuver. NC: normal control mice; COPD: PPE/LPS-induced mice; DEX: dexamethasone (3 mg/kg) + PPE/LPS-induced mice; SP: SP (50 or 100 mg/kg) + PPE/LPS-induced mice. The values are expressed as the means ± SD (*n* = 7/group). ^##^ *P* < 0.01, significantly different from NC group; ^†,††^ *P* < 0.05, *P* < 0.01, significantly different from PPE/LPS-induced group.

PPE/LPS-induced mice displayed a marked increase in the count of inflammatory cells compared to the normal controls (Figures 5A–D). However, SP treatment showed a marked reduction in inflammatory cells count, particularly, macrophages and neutrophils, in comparison to the mice with PPE/LPS-induced COPD. The levels of IL-1β, IL-6, and TNF-α significantly increased in the BALF of mice with PPE/LPS-induced COPD compared to those of the normal controls (Figures 5E–G). In contrast, SP-treated mice displayed a marked dose-dependent decrease in the levels of Th-1 cytokine compared to mice with PPE/LPS-induced COPD.

### 3.6 Effects of SP on pulmonary function of the PPE/LPS-induced COPD model

The snapshot and quick prime-3 perturbation in PPE/LPS-induced COPD mice revealed a significant decrease in the levels of total elastance (Figure 5H) and tissue elastance (Figure 5I) compared to lung tissue of the normal control. In addition, mice with PPE/LPS-induced COPD exhibited a markedly reduced FEV_0.1_/FVC value (Figure 5J) in lung tissue. However, SP-treated mice showed significantly enhanced values of total elastance and tissue elastance, and an elevated FEV_0.1_/FVC ratio in lung tissue compared with PPE/LPS-induced COPD mice.

### 3.7 Effects of SP on inflammatory cell infiltration, alveolar destruction, and collagen fiber in the lung of the PPE/LPS-induced COPD model

**FIGURE 6:**
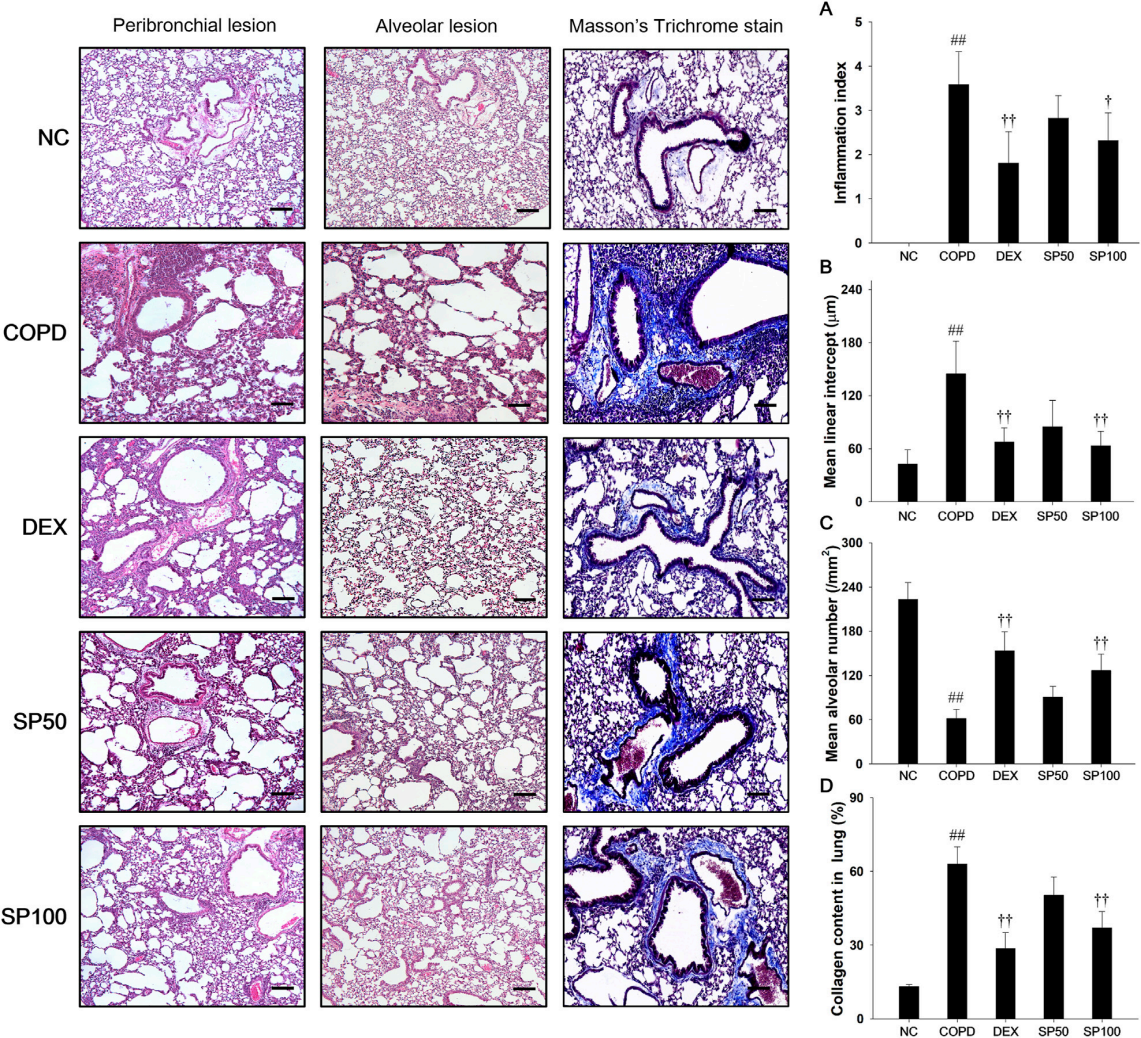
Effects of SP on inflammatory cell infiltration, alveolar destruction and collagen fiber in PPE/LPS-induced COPD model. A representative image of the airway and alveolar lesions in lung tissue stained with H&E and Masson’s trichrome staining. Quantification results of **(A)** inflammation index and alveolar destruction with airspace enlargement as **(B)** mean linear intercept and **(C)** mean alveolar number. **(D)** Collagen fiber was examined and quantified by Masson`s trichrome stain. Blue color, collagen fiber. Scale bars, 50 μm. NC: normal control mice; COPD: PPE/LPS-induced mice; DEX: dexamethasone (3 mg/kg) + PPE/LPS-induced mice; SP: SP (50 or 100 mg/kg) + PPE/LPS-induced mice. The values are expressed as the means ± SD (*n* = 7/group). ^##^ *P* < 0.01, significantly different from NC group; ^†,††^ *P* < 0.05, *P* < 0.01, significantly different from PPE/LPS-induced group.

In the lung tissue of PPE/LPS-induced COPD mice (Figures 6A–D), marked inflammatory cells infiltration into the peribronchiolar/perivascular region (Figure 6A), along with alveolar destruction, airspace enlargement (Figures 6B, C), and elevation of collagen contents (Figure 6D), were observed compared to that in lung tissue from normal control mice. However, SP treatment attenuated inflammatory cell infiltration into the peribronchiolar/perivascular region and reduced airspace enlargement with alveolar destruction. In addition, SP treatment significantly decreased the collagen contents compared to PPE/LPS-induced COPD.

### 3.8 Effects of SP on Nrf-2 pathways and oxidative stress markers in the lung tissue of the PPE/LPS-induced COPD model

**FIGURE 7:**
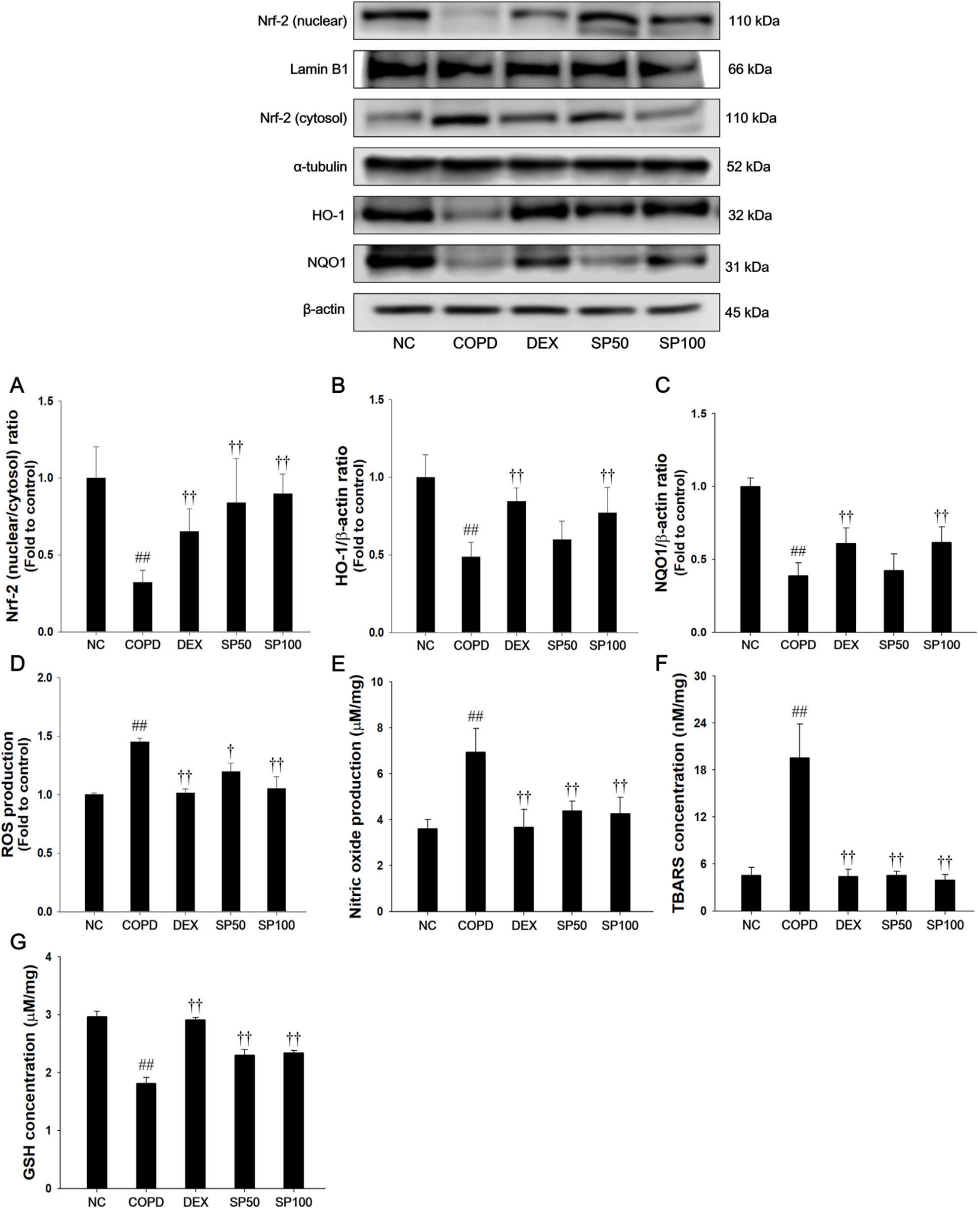
Effects of SP on Nrf-2/HO-1/NQO1 and oxidative marker in lung of PPE/LPS-induced mice. The protein expressions of **(A)** Nrf-2, **(B)** HO-1 and **(C)** NQO1 were assessed via Western blot in the lung tissue. Lamin B1, α-tubulin, and β-actin were used to confirm equal protein loading. The levels of **(D)** ROS, **(E)** NO, **(F)** TBARS, and **(G)** GSH were determined in the lung tissues. NC: normal control mice; COPD: PPE/LPS-induced mice; DEX: dexamethasone (3 mg/kg) + PPE/LPS-induced mice; SP: SP (50 or 100 mg/kg) + PPE/LPS-induced mice. The values are expressed as the means ± SD (*n* = 7/group). ^##^ *P* < 0.01, significantly different from NC group; ^†,††^ *P* < 0.05, *P* < 0.01, significantly different from PPE/LPS-induced group.

Mice with PPE/LPS-induced COPD showed a significant reduction in Nrf-2 nuclear translocation and a downregulated expression of HO-1 and NQO1 in lung tissues compared with normal control mice (Figures 7A–C). Additionally, PPE/LPS-induced COPD mice showed a significant elevation in TBARS, NO, and ROS levels with a reduction in GSH content (Figures 7D–G). However, SP treatment markedly elevated the levels of Nrf-2/HO-1/NQO1 expression. Furthermore, SP treatment markedly downregulated the levels of TBARS, ROS, and NO with increased the GSH content in lung compared with that observed in mice of PPE/LPS-induced COPD.

### 3.9 Effects of SP on TXNIP/NLRP3 inflammasome in the lung tissue of the PPE/LPS-induced COPD model

**FIGURE 8:**
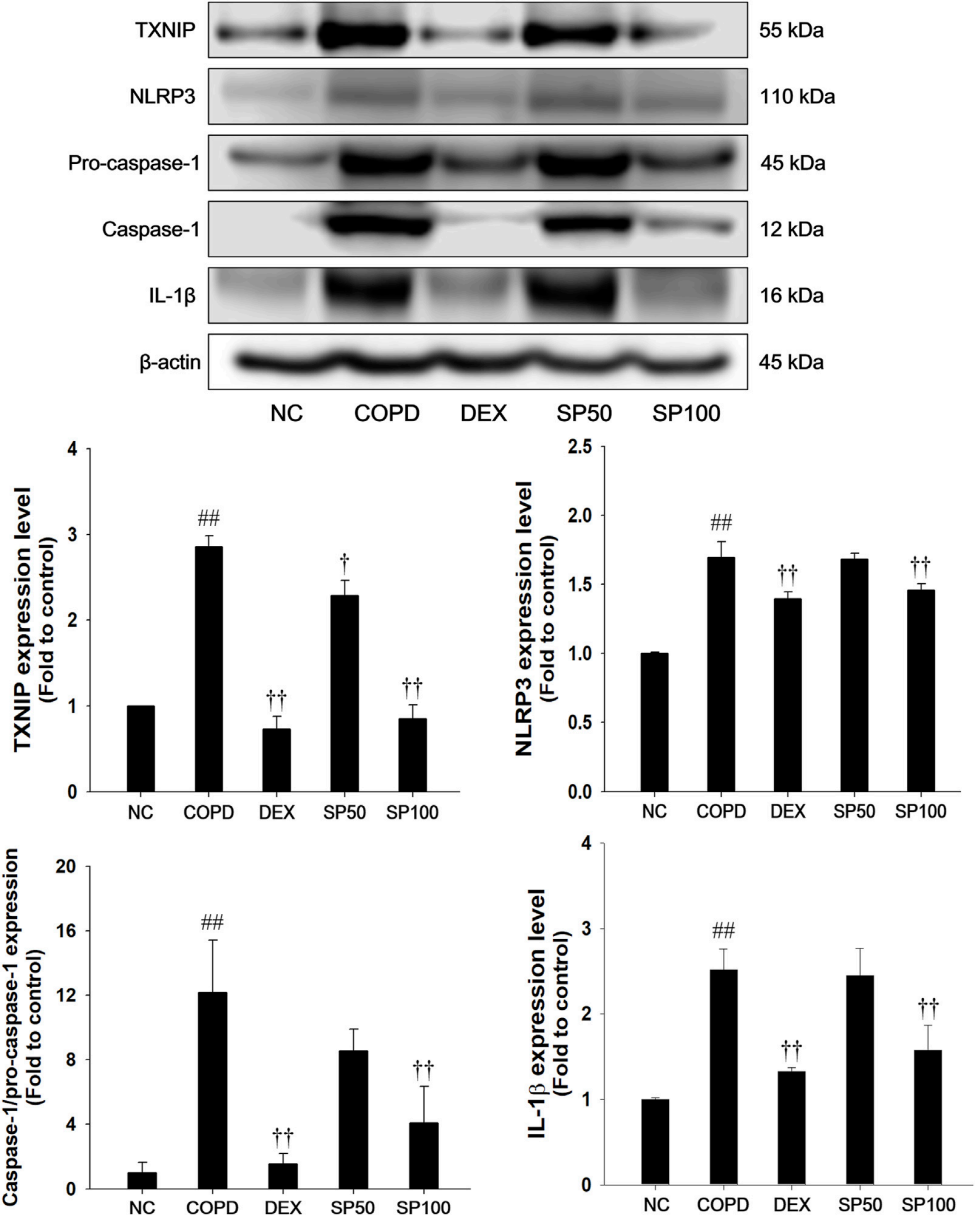
Effects of SP on TXNIP/NLRP3 inflammasome in lung of PPE/LPS-induced mice. The protein levels of TXNIP, NLRP3, pro-caspase-1, caspase-1, and IL-1β were determined by Western blot analysis in the lung tissues. β-actin was used to confirm equal protein loading. NC: normal control mice; COPD: PPE/LPS-induced mice; DEX: dexamethasone (3 mg/kg) + PPE/LPS-induced mice; SP: SP (50 or 100 mg/kg) + PPE/LPS-induced mice. The values are expressed as the means ± SD (*n* = 7/group). ^##^ *P* < 0.01, significantly different from NC group; ^†,††^ *P* < 0.05, *P* < 0.01, significantly different from PPE/LPS-induced group.

The PPE/LPS-induced COPD mice exhibited a remarkable increase in TXNIP and NLRP3 with an elevation of caspase-1 and IL-1β active forms (Figure 8). In contrast, SP treatment markedly suppressed the TXNIP and NLRP3 levels, with reduced active forms of caspase-1 and IL-1β in the lung tissues compared with those in mice with PPE/LPS-induced COPD.

## 4 Discussion

The global prevalence of COPD has raised considerably and has been a crucial problem in global health (Al Wachami et al., 2024; Zhang J. et al., 2021). COPD is triggered by prolonged exposure to chemicals, air pollutants, and cigarette smoke, and is characterized by irreversible airway limitations due to emphysema or chronic inflammation (Shin et al., 2015). Recently, novel approaches for therapeutic agents of COPD have been developed using plant-derived remedies as an alternative or complementary medicines (Kim et al., 2017a; Liao et al., 2017). In our previous study, we had shown that SP can improve antioxidant activities and attenuate inflammatory responses through upregulating of Nrf-2/HO-1/NQO1 and downregulating of MAPKs/NF-κB phosphorylation in LPS-stimulated acute lung injury model (Lee et al., 2020). In this study, we further demonstrated the effects of SP in LPS-stimulated RAW264.7 cells and PPE/LPS-induced COPD. In PPE/LPS-induced mice, SP treatment suppressed the Th-1 cytokines (IL-6, IL-1β, and TNF-α) production and reduced the number of inflammatory cells in BALF. SP administration restored total elastance, tissue elastance, and the FEV_0.1_/FVC ratio in pulmonary function tests. SP attenuated inflammatory cell infiltration with alveolar structure destruction (MLI and MAN) and reduced collagen contents in lung tissues. SP inhibited oxidative stress by suppressing ROS, NO, and TBARS levels and by stimulating Nrf-2/HO-1/NQO1 pathway. In addition, SP treatment suppressed the activation of TXNIP/NLRP3 and the subsequent inhibition of IL-1β and caspase-1 expression in the PPE/LPS-induced mice and LPS-stimulated RAW264.7 cells.

Exposure to harmful factors including particles, cigarette smoke, toxic chemicals, and bacterial toxins can activate inflammatory cells, resulting in airway inflammation in COPD (Kopa-Stojak and Pawliczak, 2024). Activated inflammatory cells, particularly macrophages and neutrophils, produce excessive ROS and release Th-1 cytokines, leading to airway inflammation (Ko et al., 2019; Xu et al., 2024). In clinical research studies, elevation of macrophage and neutrophil counts in BALF or sputum correlate with the severity airway inflammation in COPD (Baraldo et al., 2004; Guiedem et al., 2018). Moreover, activated neutrophils contribute to lung dysfunction via neutrophil elastase in COPD (Saputra et al., 2023). Neutrophil elastase induces degradation of type Ⅲ collagen and elastin, resulting in alveolar wall destruction. Alveolar wall destruction leads to irreversible enlargement of the pulmonary airspace (Devos et al., 2017; Tanaka et al., 2022). These lung structure changes are associated with pulmonary emphysema and cause pulmonary dysfunction, characterized by a reduction in total elastance, tissue elastance, and FEV/FVC ratio (Tanaka et al., 2013). The FEV/FVC ratio is a major indicator for the monitoring and diagnosis of COPD (Vogelmeier et al., 2017). In our study, SP treatment significantly inhibited the inflammatory cells counts, especially neutrophils and macrophages, and suppressed the Th-1 cytokines production in PPE/LPS-induced mice. SP-treated mice showed attenuated alveolar destruction and inflammatory cell infiltration in lung. Consistent with these results, SP effectively improved the decline in tissue elastance, total elastance, and the FEV_0.1_/FVC ratio caused by PPE/LPS instillation. It has been demonstrated that various medicines with anti-inflammatory activities significantly alleviated airway inflammation via inhibiting Th-1 cytokine production and restoring pulmonary function (total elastance, tissue elastance, and FEV/FVE ratio) in the cigarette smoke-stimulated or PPE-induced COPD models (Ko et al., 2019; Tanaka et al., 2013). Thus, these results suggest that SP treatment attenuated PPE/LPS-induced airway inflammation and pulmonary dysfunction through suppression of Th-1 cytokines and inflammatory cell infiltration.

Oxidative stress is a crucial point of lung inflammatory response in patients with COPD (Vogelmeier et al., 2017; Wang et al., 2020). Nrf-2 is an oxidant/antioxidant balance modulator and plays a key in the attenuation of inflammatory and oxidative stress-mediated diseases, such as asthma and COPD (Zhang et al., 2024). Activated Nrf-2 stimulates the transcription of phase Ⅱ antioxidant proteins, including HO-1 and NQO1. These antioxidant enzymes regulate oxidative stress via inhibition of ROS production (Lou et al., 2019). In clinical trials, patients with COPD showed reduced Nrf-2 activity in lung, resulting in decreased antioxidant activity with persistent oxidative stress and damage (Zhou et al., 2023). Nrf-2/HO-1/NQO1 signaling activation led to attenuation in oxidative stress via downregulating ROS and NO production, thereby suppressing lung inflammation in the cigarette smoke extract-stimulated COPD and PPE-induced emphysema models (Cui et al., 2017; Yomoto et al., 2020). Therefore, upregulation of the Nrf-2/HO-1/NQO1 pathway can ameliorate PPE/LPS-induced COPD. In our study, SP treatment markedly elevated Nrf-2/HO-1/NQO1 expression and inhibited ROS, NO, and TBARS levels; however, it restored GSH content in PPE/LPS-induced mice. These results indicate that the protective effects of SP on PPE/LPS-induced COPD are closely associated with the upregulating of Nrf-2/HO-1/NQO1 and its antioxidant activities.

Excessive ROS accumulation can lead to airway inflammation in COPD by activating the TXNIP/NLRP3 inflammasome (Tian et al., 2021; Qayyum et al., 2021). Under oxidative stress, TXNIP binds and directly activates the NLRP3 inflammasome (Wang et al., 2020). The activated NLRP3 inflammasome induces the IL-1β and IL-18 maturation and secretion, which amplifies the inflammatory response (Colarusso et al., 2017). In particular, IL-1β induces the production of neutrophil attractant chemokines and matrix metalloproteases, which may be related to the enhancement of the alveolar wall destruction and inflammatory cell infiltration. These results indicate that IL-1β overproduction induces airway inflammation in progression of COPD (Zhang Q. et al., 2021). SP treatment inhibited TXNIP/NLRP3 inflammasome expression with downregulation of caspase-1 and IL-1β expression in PPE/LPS-induced mice. It has been demonstrated that suppressing TXNIP/NLRP3 inflammasome can markedly repress inflammation in cigarette smoke-stimulated COPD model (Mahalanobish et al., 2020; Tian et al., 2021). Thus, these results indicate that SP ameliorates inflammatory responses by inhibiting the TXNIP/NLRP3 inflammasome pathway in mice with PPE/LPS-induced COPD.

*Spiraea prunifolia* var. *simpliciflora* (SP) has been used in traditional Korean medicine for the treatment of emetic conditions, fever, and malaria (Oh et al., 2001). In previous studies, SP showed anti-oxidative and anti-inflammatory activities in LPS-induced RAW264.7 cells (Park et al., 2013; Sim et al., 2017). *Spiraea* species were reported to contain diterpene alkaloids, diterpenes, terpenoids, glycosides, and flavonoids (Park et al., 2013). In our previous study, we demonstrated that anti-oxidative and anti-inflammatory responses of SP were related to the upregulation of Nrf-2/HO-1/NQO1 signaling-mediated suppression of MAPKs/NF-κB phosphorylation in LPS-induced acute lung injury (Lee et al., 2020). In this study, we investigated to the active components of SP, including quercetin, caffeoyl-quinic acid, and chebulinic acid. The quercetin inhibited ROS production and restored MDA, SOD, GSH, and catalase activity levels in LPS-induced rats (Huang et al., 2015). The caffeoyl-quinic acid downregulates expression of Th-1 cytokines and NO production in LPS-induced RAW264.7 cells (Kim et al., 2019). The chebulinic acid suppressed IL-6, TNF-α, and ROS via inhibition of NF-κB pathway in LPS-induced inflammatory bone loss mice model (Sharma et al., 2023). These results provide evidence that SP can be used to treat COPD. However, major compounds of SP have not been determined for their anti-oxidant and anti-inflammatory activities in PPE/LPS-induced COPD model. Thus, further analysis using a PPE/LPS-induced COPD model will reveal to curative effects.

## 5 Conclusion

To the best of our knowledge, the finding of the present study imply that SP can effectively attenuate ROS-mediated oxidative stress and airway inflammation in mice with PPE/LPS-induced COPD. This likely occurs via upregulation of Nrf-2/HO-1/NQO1 and suppression of TXNIP/NLRP3 inflammasome pathways. Thus, our study provides evidence that SP could have valuable therapeutic potential for the treatment of COPD.

## Data Availability

The raw data supporting the conclusions of this article will be made available by the authors, without undue reservation.
